# Excitation of optical tamm state for photonic spin hall enhancement

**DOI:** 10.1038/s41598-023-50067-7

**Published:** 2024-01-02

**Authors:** Amit Kumar Goyal, Divyanshu Divyanshu, Yehia Massoud

**Affiliations:** Innovative Technologies Laboratories (ITL), King Abdullah University of Science and Technology (KAUST), Thuwal, 23955 Saudi Arabia

**Keywords:** Nanophotonics and plasmonics, Photonic crystals, Optical sensors, Nanophotonics and plasmonics, Nanosensors

## Abstract

This work presents a dielectric material-based optical Tamm state (OTS) excitation technique with modified dispersion characteristics for photonic spin hall effect (PSHE) enhancement. The dispersion analysis of the structure is carried out to validate OTS’s localization and corresponding PSHE generation for a given polarization at 632.8 nm incident wavelength. The exceptional points are optimized by considering thickness-dependent angular dispersion analysis. PSHE-based transverse displacement (PSHE-TD) is dependent on the defect layer thickness. The optimized structure provides 10.73 $\times \lambda$ (or 6.78 $\upmu$m) PSHE-TD at an incidence angle of 41.86${}^{\circ }$. The PSHE-TD of the optimized structure is sufficiently high due to the much narrower resonance than the plasmonic-based structures. Further, the structure’s potential to function as a PSHE-TD-based optical sensor is assessed. The optimized structure shows an analytical average sensitivity of about 43,789 $\upmu$m/RIU showing its capability to detect the analytes with refractive index variations in the $10^{-4}$ range. The structure demonstrates a three-time sensitivity improvement compared to similar resonance designs. Considering only dielectric materials in the proposed structure and considerably enhanced PSHE-TD, the development of highly efficient PSHE-TD-assisted commercial structures is anticipated.

## Introduction

When incoming light travels through an inhomogeneous medium or an optical interface, the photonic spin-hall effect (PSHE) occurs because of a transverse spin-assisted shift relative to the geometric optical trajectory of the photons^1–5^. The PSHE considered as an optical analogue to the spin Hall effect in an conventional electronic system. Where the photons spin is considered and takes the role of the electrons spin, and the refractive index gradient takes the place of the applied electric field. A detailed theoretical technique for estimating the PSHE was presented by Bliokh et al.^6^. Further, the origin of PSHE was established with spin–orbit interaction (SOI) of light, orbital angular momentum, and the geometric phases, i.e., Pancharatnam–Berry (PB) phase and Rytov–Vlasimirskii (RV) phase^7^. PSHE effect leads to the splitting of the reflected wave into its polarized states (LCP/RCP or V/H polarization). Generally, the PSHE effect based transverse displacement is very small that cannot be detected using direct optical measurement. Thus a specific weak measurement technique is utilized to measure the small PSHE shift. This effect was first experimentally demonstrated by Hosten et al.^8^ in 2008 using weak measurement technique^9^, which utilized pre-selection and post selection method. This motivated research towards obtaining enhanced PSHE-based shift in different materials and optical systems^10–15^.

Several nanophotonic techniques such as Brewster angle^16^, optical pumping^17^, lossy mode resonance (LMR)^18^, and surface plasmon resonance (SPR)^19^, etc. are also proposed to enhance and control the PSHE. Out of these techniques, SPR-based enhancement techniques have been widely investigated for enhancing the PSHE effect^19,20^. Zhou et al.^19^ used the surface plasmon resonance (SPR) effect to enhance the PSHE transverse displacement (PSHE-TD) in the year 2016. Further, Brewster’s angle concept in SPR has also been utilized to enhance PSHE-TD. At this angle the device shows a reduced Fresnel reflective coefficient and hence enhanced PSHE-TD^21^. However, the higher absorption losses limits its performance gain significantly, causing inferior resonance metrics. Moreover, the SPR effect is stimulated only at a constant working wavelength, specifically for longitudinal modes (TM modes)^22^. Thus, it is not possible to generate and enhance PSHE for both the polarization.

Recently, optical Tamm state (OTS) or Bloch surface mode (BSW) has been explored as an alternative to the SPR mode, which can be excited by considering a dielectric material-based one-dimensional photonic crystal (1D-PhC) structure^23–25^. This OTS/BSW mode possesses similar characteristics to the SPR mode, which can then be excited for both the polarization’s^26,27^. Additionally, owing to the ultra-sharp optical and better resonance metrics in BSW compared to SPR^28,29^, a larger value of the Fresnel reflection ratio is possible. Therefore, this work investigates the dielectric 1D-PhC-based OTS excitation optimization toward PSHE-TD enhancement. The dispersion characteristics of the proposed 1D-PhC structure is tailored by considering a top defect layer of low index material. The OTS in optimized dielectric1D-PhC exhibits better resonance performance than its corresponding metallic SPR counterparts. The proposed structure demonstrates robust OTS localization for an optimized 155 n$\textrm{m}$ top layer thickness. Furthermore, the impact of defect layer thicknesses on PSHE-TD generation by OTS propagation is investigated. This gives a considerably higher reflection ratio for *p*-polarized and *s*-polarized light, which is crucial for attaining a higher PSHE-TD value. The theoretical results exhibit a 10.73 $\times \lambda$ (or 6.78 $\upmu$m) improved PSHE-TD at an incidence angle of 41.86${}^{\circ }$. Owing to improved PSHE-TD, the structure’s sensing capability using the PSHE-based interrogation method is also demonstrated. The PSHE-TD interrogation method exhibits a 43,789 $\upmu$m/RIU sensitivity, which shows a $\approx$ 224% improvement than the corresponding Lossy mode resonance structure^18^. Moreover, the structural sensitivity performance is much better than the recent reported sensors^30–34^. Finally, a comparative performance matrix is provided with the similar reported works. This shows that the proposed device gives better performance having a simpler structure, easier fabrication, and low cost possibilities.

The work is categorized in the following sections as follows: proposed 1D-PhC structure for OTS excitation and its analytical model for PSHE generation is given in “Device structure and analytical model”. Impact of top defect layer optical thickness and corresponding PSHE-TD enhancement is presented in “Results and discussion”, and the work is concluded in “Conclusion”.

**Figure 1:**
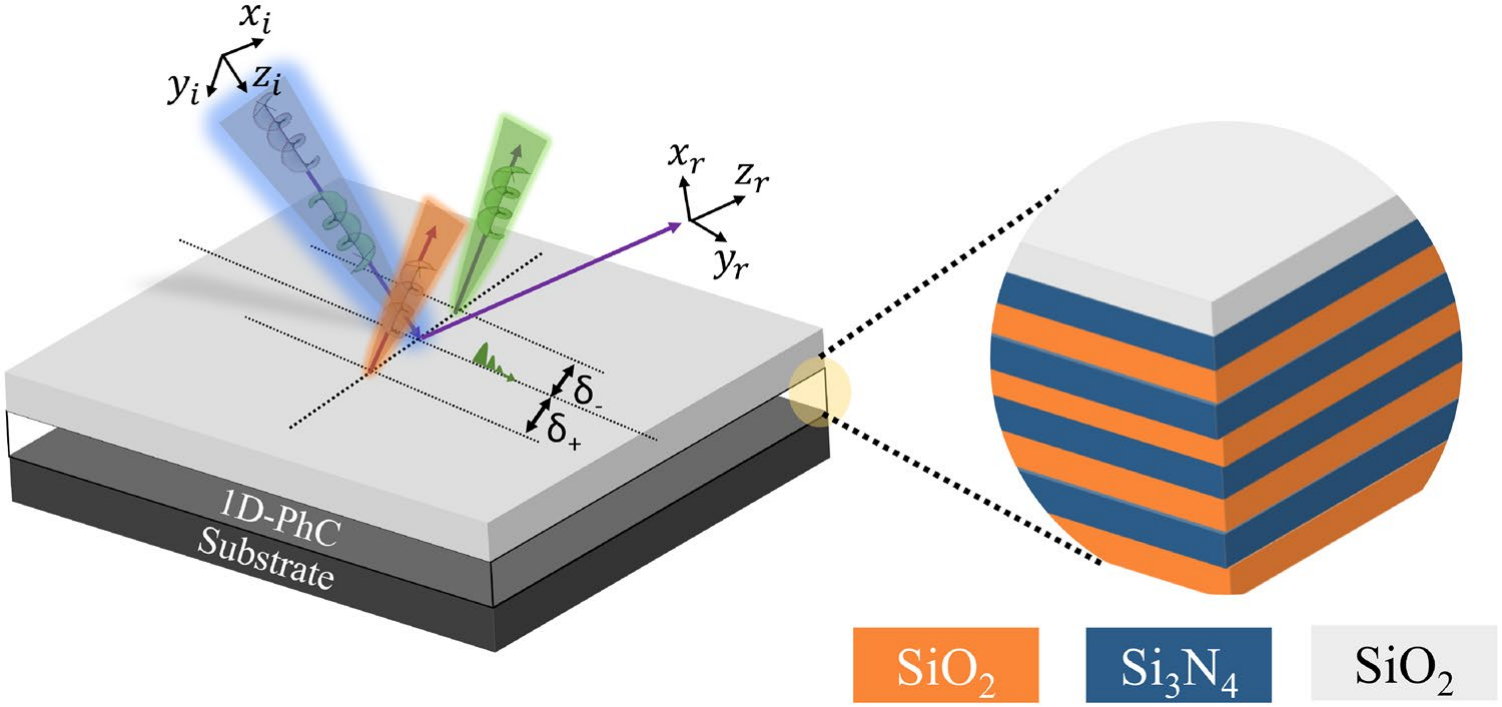
The schematics of induced PSHE-TD because of OTS localization at the top layer of the 1D-PhC structure [Substrate (Glass) | ($A_{\text {SiO}_{\text {2}}}$, $B_{\text {Si}_{\text {3}}\text {N}_{\text {4}}}$)$^N$ | D | Air].

## Device structure and analytical model

The schematics of the proposed 1D-PhC device and corresponding induced PSHE-TD at the top dielectric-air interface of the proposed structure is represented in Fig. 1. The devices consists of [Substrate (Glass) | ($A_{\text {SiO}_{\text {2}}}$, $B_{\text {Si}_{\text {3}}\text {N}_{\text {4}}}$)$^N$ | defect (D) | Air] configuration. The structure is a periodically alternating layers of materials ‘A’,‘B’, with low RI ($n_{L}$) and higher RI ($n_{H}$), respectively. The structural symmetry is deliberately broken by considering a defective low-index material top layer (D). The proposed device is uniform in x-direction and periodic in the normal z-direction, with periodicity ($\Lambda$) of ($A_{\text {SiO}_{\text {2}}}$, $B_{\text {Si}_{\text {3}}\text {N}_{\text {4}}}$) repeated ‘N’ times.

Hence, the refractive index profile in the normal z-direction $n(z+\Lambda )=n(z)$ can be calculated by Eq. (1) as:

1 $$\begin{aligned} RI(n(z))=\left\{ \begin{array}{ll} RI(n_L),&{} 0<z<A_t \\ RI(n_H),&{} A_t<z<A_t+B_t \end{array} \quad \textit{with N= 7}\right. \end{aligned}$$

Here, $A_{t}$ and $B_{t}$ are the ‘A’ and ‘B’ layer’s physical thicknesses. The Helmholtz equation is utilized further to calculate the electric field confinement within the structure^35^. To establish the dispersion relation for the OTS and to solve the eigenvalue problem, the Floquet theorem is employed^36^:

2 $$\begin{aligned} K(\beta , \omega )=\frac{1}{\Lambda } \cos ^{-1}\left( \frac{1}{2}\left( M_1+M_4\right) \right) \end{aligned}$$

Here, $M_{n}$ represents the eigenvalue matrix elements, whereas the Bloch wave vector is represented by ‘K’. The real values of Bloch wave vector attributes to the propagating OTS, and imaginary values give information on evanescent OTS. Furthermore, the transfer matrix is formulated to calculate the reflected and transmitted wave amplitude.

When a monochromatic Gaussian beam of wavelength $\lambda$ having beam waist of $w_{0}$ is incident at the proposed device, the angular spectrum is represented by Eq. (3). The PSHE effect divides this incident Gaussian beam into two corresponding circularly polarized components.

3 $$\begin{aligned} \tilde{{\textbf{E}}}_{i \pm }=\left( {\textbf{e}}_{i x} \pm i{\textbf{e}}_{i y}\right) \frac{w_0}{\sqrt{2 \pi }} \exp \left[ -\frac{w_0^2\left( k_{i x}^2+k_{i y}^2\right) }{4}\right] , \end{aligned}$$

here, $k_{iy}$ and $k_{ix}$ are the wave-vector components in the $y_{i}$ and $x_{i}$ direction, and $+/-$ represents the corresponding left and right circular polarization components. The spin-basis set of incident Gaussian beam is represented in Eq. (4),

4 $$\begin{aligned} \tilde{{\textbf{E}}}_i^V=i\left( \tilde{{\textbf{E}}}_{i-}-\tilde{{\textbf{E}}}_{i+}\right) / \sqrt{2} {, } \tilde{{\textbf{E}}}_i^H=\left( \tilde{{\textbf{E}}}_{i+}+\tilde{{\textbf{E}}}_{i-}\right) / \sqrt{2} \end{aligned}$$

where *V* and *H* represents the vertical and horizontal polarization states. Further, the mathematical relations in incident and reflected angular spectra is given by Eq. (5)^16^,

5 $$\begin{aligned} \tilde{\textrm{E}}_r\left( k_{r x}, k_{r y}\right) =\mathrm {M_{R}} \tilde{\textrm{E}}_i\left( k_{i x}, k_{i y}\right) \end{aligned}$$

where

$$\mathrm {M_{R}}=\left[ \begin{array}{cc} r_p &{} \frac{k_{r y} \cot \theta _i\left( r_s+r_p\right) }{k} \\ -\frac{k_{r y} \cot \theta _i\left( r_s+r_p\right) }{k} &{} r_s \end{array}\right]$$

Here, ‘$M_{R}$’ is the rotational matrix.

Utilizing Eqs. (4)–(5), the reflected angular spectrum is calculated and the Fresnel reflection coefficient is calculated by utilizing Taylor series expansion in transfer matrix method (TMM)^37^,

6 $$\begin{aligned} r_{s, p}\left( k_{i x}=0\right) +\sum _{j=1}^n \frac{k_{i x}{ }^n}{j !}\left[ \frac{\partial j_{r_{s, p}}\left( k_{i x}\right) }{\partial k_{i x}{ }^j}\right] _{k_{i x}=0} \end{aligned}$$

The Eq. (6) is used to determine the PSHE-TD shift with regard to geometrical optic prediction^19^,

7 $$\begin{aligned} \delta _{\pm }^{V, H}=\frac{\iint \tilde{{\textbf{E}}}^* i \partial _{k_{r y}} \tilde{{\textbf{E}}} d k_{r x} d k_{r y}}{\iint \tilde{{\textbf{E}}}^* \tilde{{\textbf{E}}} d k_{r x} d k_{r y}}. \end{aligned}$$

Further, considering first-order Taylor-series expansion approximation to expand the Fresnel coefficients of Eq. (6) and using Eqs. (4)–(7), $\delta _{\pm }^{V}$ is obtained and is represented in (8)^20^:

8 $$\begin{aligned} \delta _{\pm }^{V}=\mp \frac{k w_0^2 \textrm{R} e\left( 1+r_p / r_s\right) \cot \theta _i}{k^2 w_0^2+\left| \frac{\partial l n r_s}{\partial \theta _i}\right| ^2+\left| \left( 1+r_p / r_s\right) \cot \theta _i\right| ^2}, \end{aligned}$$

The PSHE based transverse displacement $\delta _{+}$ possesses the similar magnitude of $\delta _{-}$. Therefore, only $\delta _{-}^{V}$ is considered here for further calculation and the same results can be obtained for $\delta _{+}^{V}$ .

**Figure 2:**
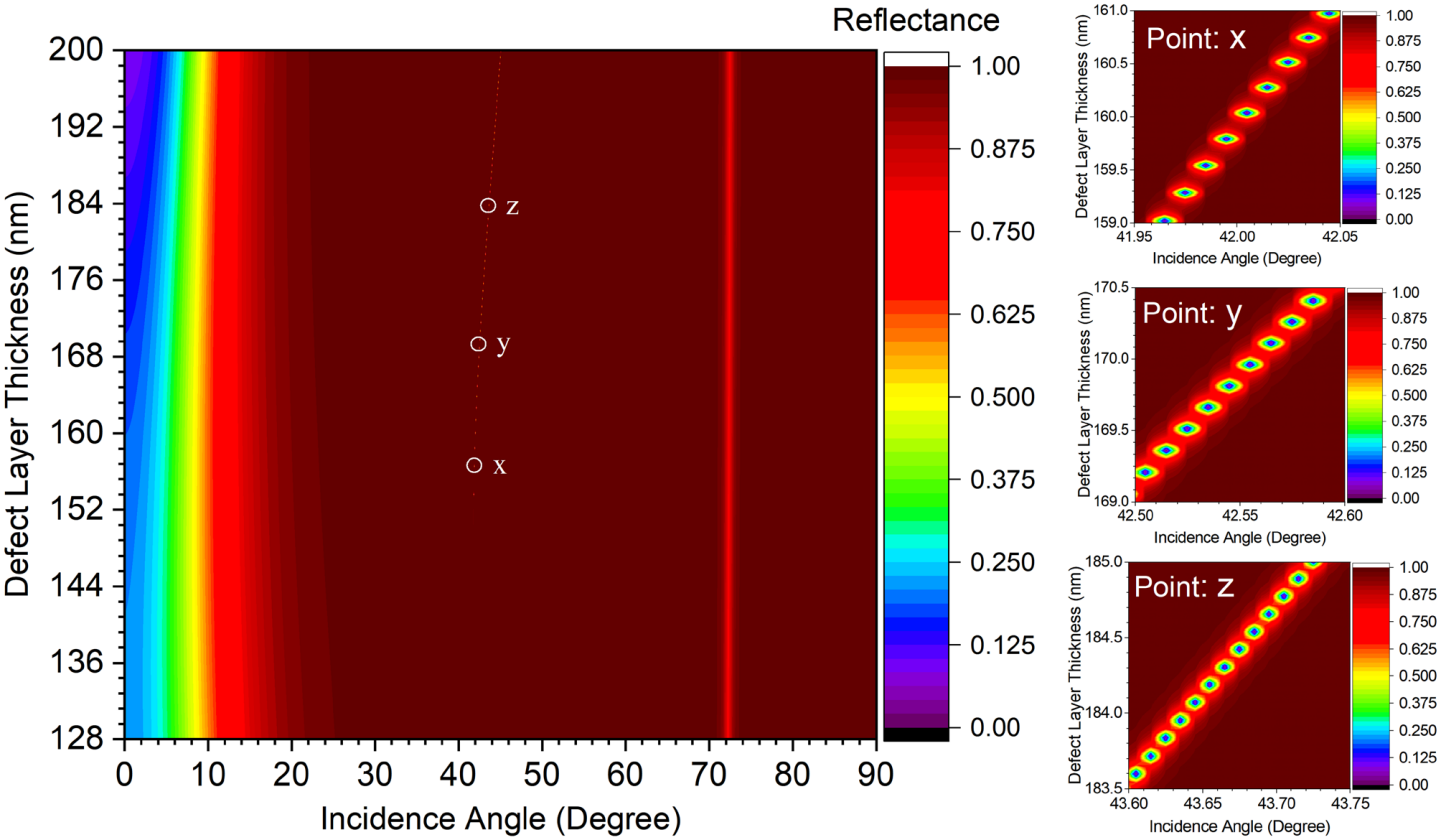
Angular dispersion analysis for optical Tamm state (OTS) localization at the top layer of proposed 1D-PhC [Substrate (Glass)|($A_{\text {SiO}_{\text {2}}}$, $B_{\text {Si}_{\text {3}}\text {N}_{\text {4}}}$)$^N$ | D| Air].

## Results and discussion

**Figure 3:**
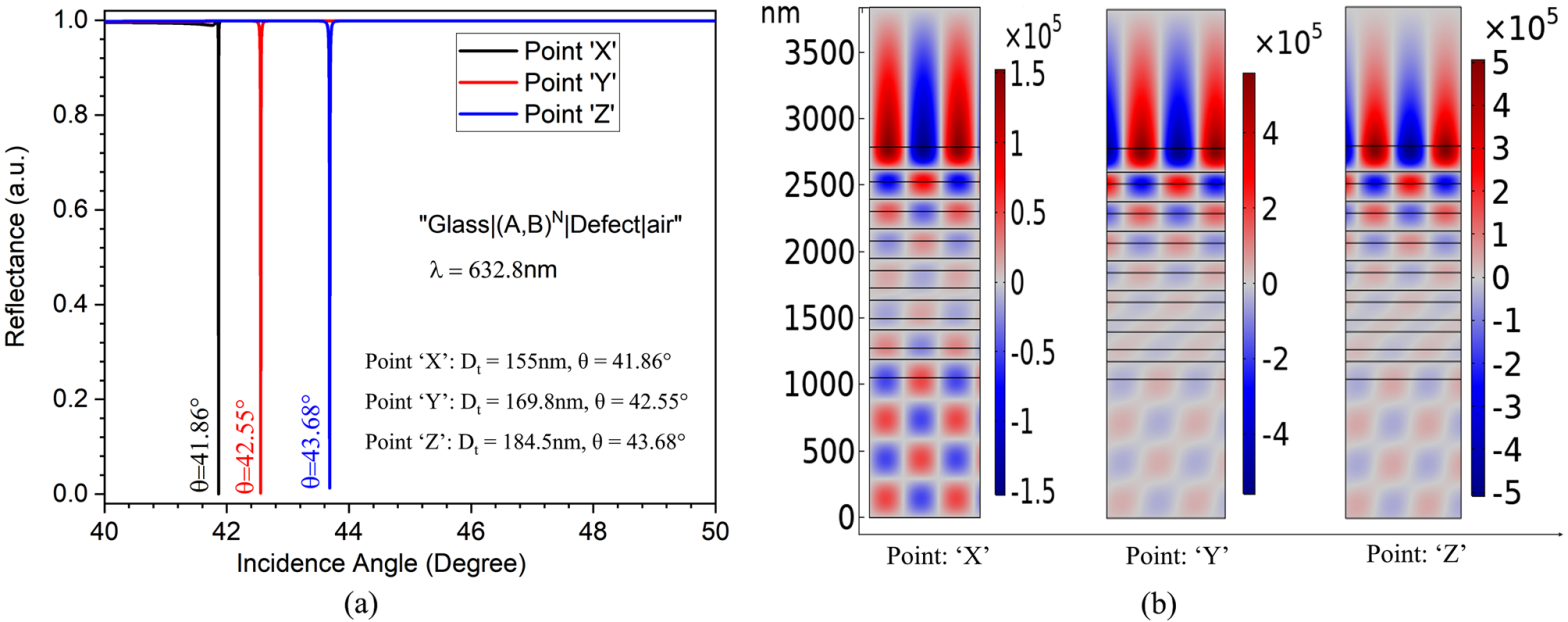
(**a**) Reflectance response of proposed 1D-PhC deice, and (**b**) corresponding OTS localization and electric field distribution for considered ‘X’, ‘Y’, and ‘Z’ points.

The 1D-PhC [Substrate(Glass)| ($A_{\text {SiO}_{\text {2}}}$, $B_{\text {Si}_{\text {3}}\text {N}_{\text {4}}}$)$^N$ | D | Air] comprises $A_{t}$ = 128 n$\textrm{m}$, $B_{t}$ = 85 n$\textrm{m}$, $n_{L}$ = 1.46, $n_{H}$ = 2.2, $k_{A}$ = 0, and $k_{B}$ = -0.0002. Initially, the impact of top layer thickness on OTS excitation for TE polarization and corresponding PSHE generation is investigated. Thus, the defect layer thickness-dependent, angular dispersion analysis of the 1D-PhC is represented in Fig. 2. The device demonstrates a cutoff defect layer thickness of about 150 n$\textrm{m}$ beneath that, no OTS is excited. This is because the dispersion curve for OTS with 150 n$\textrm{m}$ defect layer thickness approaches the air light line for operating wavelength (632.8 n$\textrm{m}$). However, a strong OTS is excited beyond this point, as shown in Fig. 2. The OTS excitation characteristics at the top dielectric-air interface is investigated at three defect layer thicknesses, shown as ‘X’, ‘Y’, and ‘Z’ in Fig. 2. With an incident wavelength of 632.8 n$\textrm{m}$, point ‘X’ has $D_{t}$ = 155 n$\textrm{m}$, and $\theta _{i}$ $\approx$ 41.86${}^{\circ }$, point ‘Y’ is $D_{t}$ = 169.8 n$\textrm{m}$, and $\theta _{i}$ $\approx$ 42.55${}^{\circ }$ and point ‘Z’ is $D_{t}$ = 184.5 n$\textrm{m}$, and $\theta _{i}$ $\approx$ 43.68${}^{\circ }$, respectively. The structural reflectance response at considered three points is shown in Fig. 3. Thus, for $D_{t}$ $\in$ (150 n$\textrm{m}$, 200 n$\textrm{m}$), $\theta _{i}$ $\ge$ $\theta _{b}$ (Critical angle $\approx$ 41${}^{\circ }$) OTS propagation is sustained.

From Eqs. (7)–(8), it can be shown that the PSHE-TD shift is a function of $\frac{\mid r_{TE}\mid }{\mid r_{TM}\mid }$ (or $\frac{\mid r_{TM}\mid }{\mid r_{TE}\mid }$) depending on the considered TE (or TM) polarization. The reflectance response at the three considered points ‘X’, ‘Y’, and ‘Z’ is illustrated in Fig. 3a. The 1D-PhC structure shows a significantly higher reflection for TM polarization and very low value for TE-polarization. This gives a very high $\frac{\mid r_{TM}\mid }{\mid r_{TE}\mid }$ or $\frac{\mid r_{p}\mid }{\mid r_{s}\mid }$ for the selected points. Furthermore, the OTS confinement and electric field distribution analysis for these ‘X’, ‘Y’, and ‘Z’ points is presented in Fig. 3b. This is calculated using the finite element method (FEM) of COMSOL Multiphysics. The device exhibits a strong OTS localization for higher defect layer thickness (having normalized electric field intensity of around 5.5$\times$ 10$^{5}$ V/m). Decreasing defect layer thickness exhibits higher evanescent mode coupling in the air region (having normalized electric field intensity of around 1.52 $\times$ 10$^{5}$ V/m). Thus, by choosing proper incident wavelength and top layer thickness, the proposed device can also be used for both sensing (low thickness) and wave-guiding (high thickness) applications. The structure can generate enhanced PSHE-TD at all the defect layer thicknesses beyond the cut-off value (at different $\theta _{i}$).

**Figure 4:**
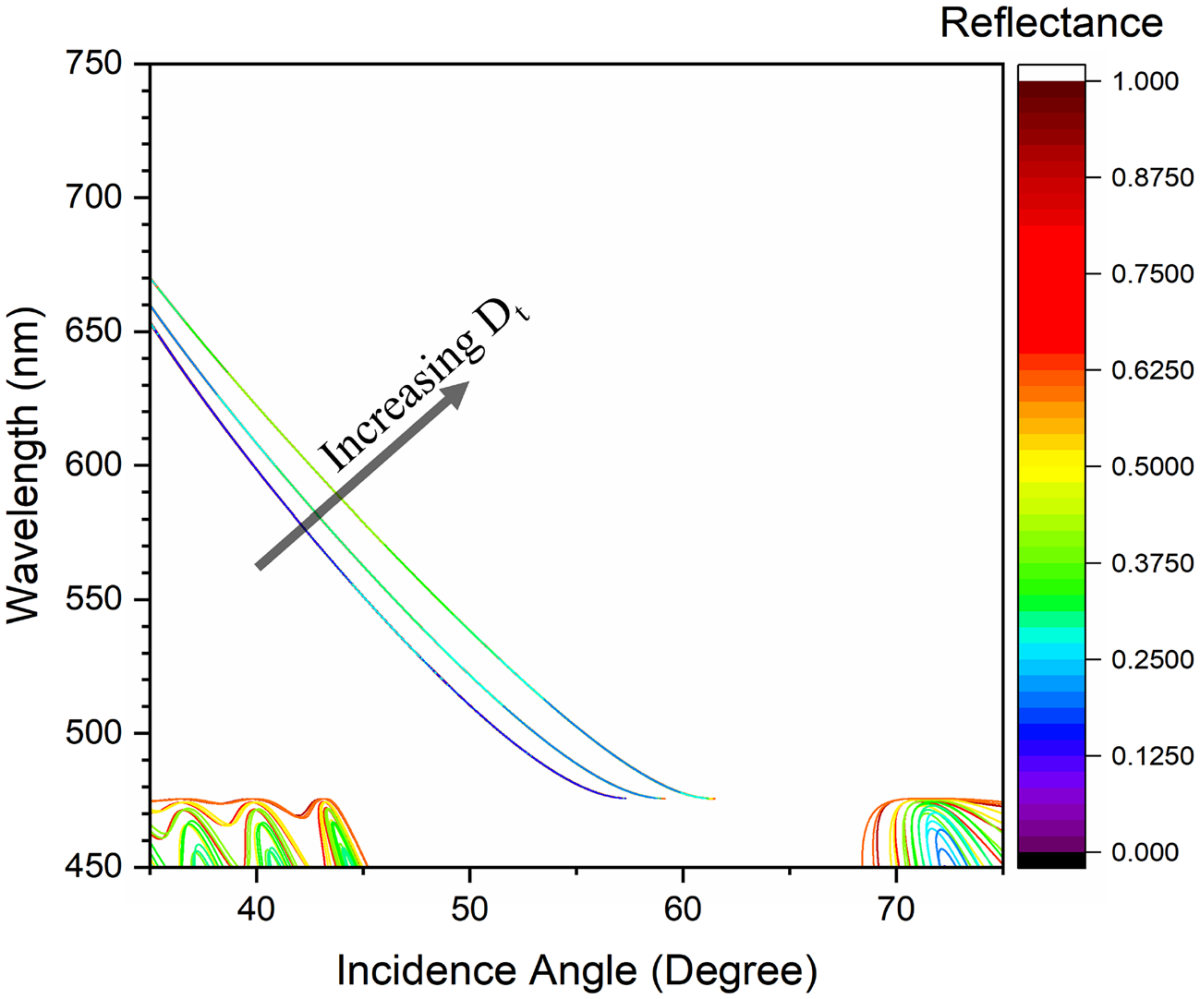
The defect layer thickness-dependent angular dispersion for TE polarisation at $\lambda$ = 632.8  n$\textrm{m}$.

It is noteworthy to mention that the proposed structure is optimized to excite the OTS at a 632.8 n$\textrm{m}$ incident wavelength. However, the structure can also be optimized to excite the OTS at other wavelengths. Figure 4 represents the wavelength dispersion analysis of the 1D-PhC for a given defect layer thickness. This demonstrates the structure’s capability to excite an OTS at any user-defined wavelength by just changing the defect layer thickness. After OTS excitation, the structure capability to enhance PSHE-TD is evaluated. Thus, with $\lambda$ = 632.8 n$\textrm{m}$ and $D_{t}$ = 155 n$\textrm{m}$, $\theta _{i}$ $\approx$ 41.86${}^{\circ }$, PSHE-TD analysis is further analyzed. The structure is showing a extremely small full-width-half-maximum (FWHM) at $\frac{\mid r_{TE}\mid }{\mid r_{TM}\mid }$ $\approx$ 0.5 of around 0.003${}^{\circ }$. This shows a very narrow region for producing higher $\frac{\mid r_{TM}\mid }{\mid r_{TE}\mid }$ based on $\theta _{i}$. The higher reflection sensitivity towards narrower angle dependency exhibits its potential applications in both sensing and precision metrology. Figure 3a, shows that for small $\partial$ $\theta _{i}$ at 41.86${}^{\circ }$, the term $\left| \frac{\partial l n r_s}{\partial \theta _i}\right| ^2$ $\approx$ 0.

**Figure 5:**
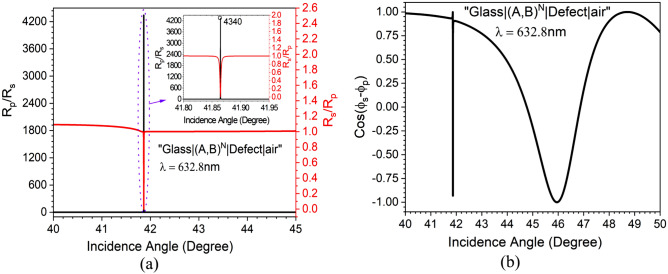
(**a**) Incident angle-dependent Fresnel reflectance coefficients ratio variations, and (**b**) cosine of phase difference with variation in the incident angle for 632.8 n$\textrm{m}$ wavelength.

Thus, the zeroth-order Taylor series expansion of Eq. (6) can be used to obtain $\delta _{\pm }^{V}$. This leads to a simplified expression for PSHE calculation with sufficient accuracy^11,38,39^:

9 $$\begin{aligned} \delta _{\pm }^{V}=\mp \left( 1+{\text {Re}}\left[ r_p\right] / {\text {Re}}\left[ r_s\right] \right) \cot \theta _{i} / k \end{aligned}$$

Further, for an incidence angle of 41.86${}^{\circ }$ and $\lambda$ = 632.8 n$\textrm{m}$, the structure exhibits a very high reflectance ($\mid r_{TM}\mid$) for *p*-polarized and almost negligible reflection ($\mid r_{TE}\mid$) for *s*- polarized light, which is shown in Fig. 3. Thus, a significantly higher value of $\frac{\mid r_{TM}\mid }{\mid r_{TE}\mid }$ of around 4340 is obtained around the resonance angle ($\theta _{r}$). This is represented in Fig. 5a. Since $\delta _{\pm }^{V}$ also depends on both Fresnel coefficient phases ($\phi _{s}$,$\phi _{p}$) and cos($\phi _{s}$-$\phi _{p}$), therefore $\theta _{i}$ dependent ($\phi _{s}$,$\phi _{p}$) and cos($\phi _{s}$-$\phi _{p}$) values are calculated and are illustrated in Fig. 5b. An sharp change in the $\mid$cos($\phi _{s}$-$\phi _{p}$)$\mid$ is obtained at resonance angle, which is generally the observed case for enhanced PSHE-TD generation approach.

**Figure 6:**
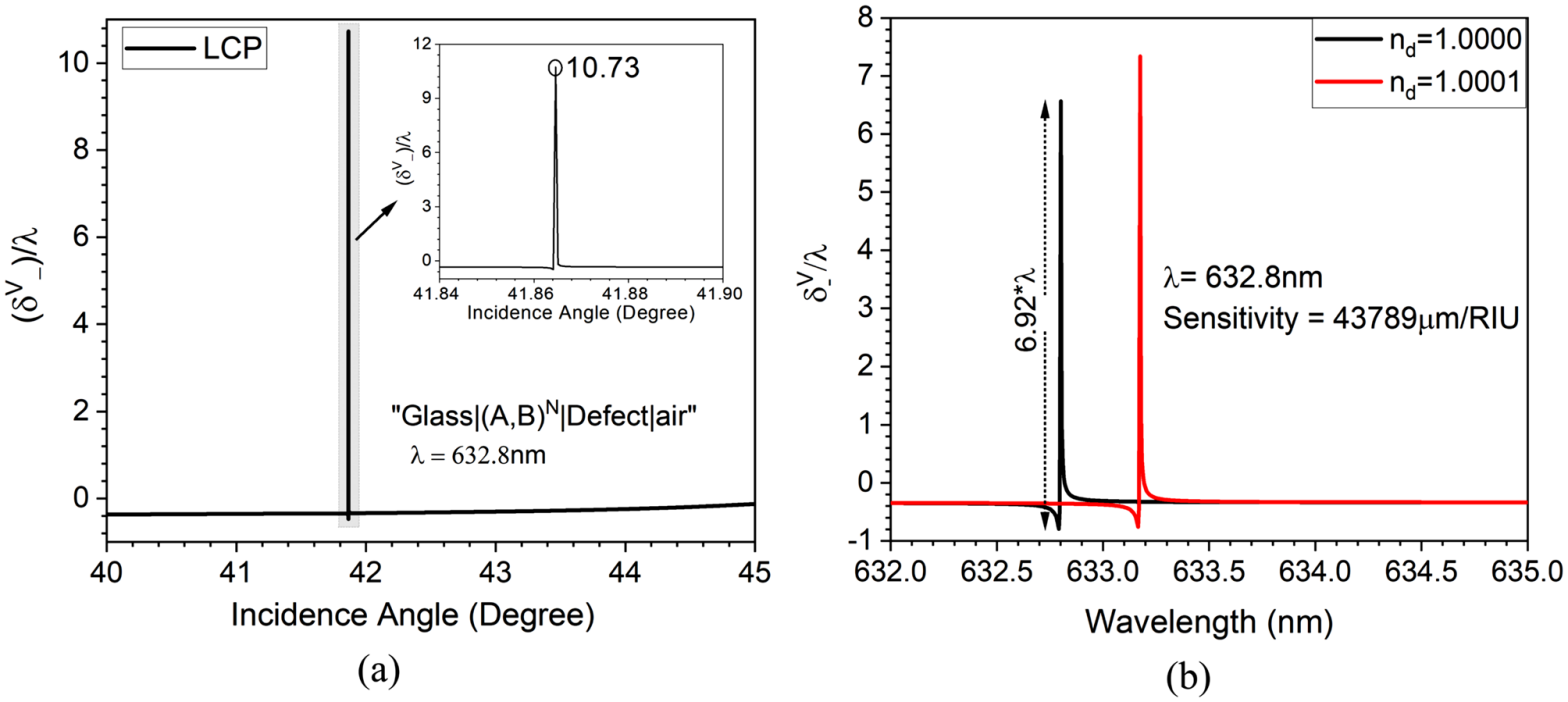
(**a**) The PSHE-TD at 41.86${}^{\circ }$ incident angle for the optimized structure at 632.8 n$\textrm{m}$ wavelength, and (**b**) the PSHE-TD-based sensitivity performance analysis.

Finally, PSHE-TD is calculated for the proposed optimized structure. The calculated PSHE-TD for *V* polarized light normalized to wavelength is shown in Fig. 6a. The optimized parameters exhibit a maximum PSHE-TD of 10.73$\times \lambda$ at $\theta _{r}$ = 41.86${}^{\circ }$. This leads to a total PSHE-TD of around 6.78 $\upmu \textrm{m}$ for the proposed structure. The obtained $\Delta \delta _{-}^V$ also demonstrates a narrower FWHM of around 0.003${}^{\circ }$.

Further, PSHE-based wavelength interrogation is utilized to demonstrate the PSHE-assisted sensing capability of the proposed structure. For sensing, an analyte with different dielectric constants is infiltrated at the top interface, which changes the top interface’s effective RI. Thus, change in both PSHE-TD and operating wavelength at a given incidence angle is observed, which is used to calculate the devices’ sensitivity.

The PSHE-TD based sensitivity ($S_{TD}$) values for constant wavelength ($\lambda$) and incidence angle is analytically obtained using the PSHE-TD ($\Delta \delta _{-}^V$) shift and is given by the relation:

10 $$\begin{aligned} \left. S_{T D}^V\right| _{\lambda = constant}=\frac{\Delta \delta _{-}^V}{\Delta n_d} \end{aligned}$$

Here $\Delta n_{d}$ represents the difference in the infiltrated analyte’s RI. The proposed 1D-PhC is highly sensitive, and can detect a very small perturbation at the top interface. Therefore, the structure’s sensitivity is measured by considering a 0.0001 variation in the RI (1.000–1.0001) at the top interface. This exhibits PSHE-TD shift ($\Delta \delta _{-}^V$) of around 6.92$\lambda$ (6.565$\lambda$ at 1.000 and − 0.356$\lambda$ at 1.0001) for respective RI variation ($\Delta$ $n_d$) of $10^{-4}$. This provides an average $S_{T D}^V$ of about 43,789 $\upmu \textrm{m}$/RIU, illustrated in Fig. 6b. The obtained sensitivity is much higher than the previously reported 1D-PhCR and SPR-based PSHE sensors^39–42^. Finally, the PSHE-based sensitivity of the 1D-PhC optimized structure is compared with the recently reported similar SPR and LMR-based structure and is reported in Table 1. In comparison to previously reported PSHE-TD sensors, the optimized 1D-PhC exhibits significantly improved PSHE-TD value, resulting in substantially better sensitivity. Additionally, the structure demonstrates its capability to detect a minute $10^{-4}$ fluctuation in the higher RI range (1.0–1.5). Furthermore, the design can be fabricated by considering deposition or dip/spin coating techniques^43^. The proposed structure has several advantages due to its simple dielectric materials-based structure, easier fabrication and characterization with low optical losses compared with various 2D and 3D devices based on metasurfaces,and meta lenses.

**Table 1:** PSHE-TD and sensing performance comparison of proposed structure with recently reported literature.

Device	Materials	Wavelength	PSHE-TD	$$\begin{array}{c} \text{ Sensitivity } \\ (\upmu \textrm{m}/\textrm{RIU})\end{array}$$	RI sensing range	Year
LMR	ITO and water	1151.9 n$\textrm{m}$	13.28 $\upmu \textrm{m}$	13,500	1.3330–1.3340	2022^18^
SPR	Sodium, PMMA, and graphene	1200 n$\textrm{m}$	0.325 $\upmu \textrm{m}$	1844.9	1.3195–1.3460	2021^41^
SPR	Silver and gold	632.8 n$\textrm{m}$	5.34 $\upmu \textrm{m}$	6602	1.4580–1.4590	2018^11^
SPR	Gold and graphene	633 n$\textrm{m}$	$\approx$ 0.09 $\upmu \textrm{m}$	$10^5$ (amplified)	1.3300–1.3350	2018^39^
OTS	$$\begin{array}{c}\text {Substrate}\,\mid \,(A,\,B)^{N}\,\mid \,\text {Defect}\,\mid \,\text {Air } \\ \text {A, Defect}\,=\,\text {SiO}_{\text {2}},\text {B}\,=\,\text {Si}_{\text {3}}\text {N}_{\text {4}}\\ A_{t}: 128 nm, D_{t}: 155 nm, B_{t}: 85 nm \end{array}$$	632.8 n$\textrm{m}$	6.78 $\upmu \textrm{m}$	43,789	1.000–1.0001	$$\begin{array}{c}\text {This} \\ \text {Work} \end{array}$$

## Conclusion

The work presents an optical Tamm state (OTS)-assisted PSHE generation and corresponding PSHE-TD enhancement. The work focused on optimizing top layer’s optical thickness parameters to generate and localize OTS and corresponding PSHE generation. The impact of working wavelength, incidence angle and optical thickness of top interface layer is thoroughly investigated to enhance the PSHE-TD. The analytical results demonstrate the confinement of the OTS for 632.8 n$\textrm{m}$ wavelength providing a maximum PSHE-TD of around 6.78 $\upmu \textrm{m}$ (10.73 $\times \lambda$). Finally, the structure’s capability to detect a minute change in the surrounding analyte refractive index is demonstrated. The analytical results exhibit a 43,789 $\upmu \textrm{m}$/RIU average sensitivity with a 0.0001 change in the analyte refractive index unit. This demonstrate its potential to detect the analyte having refractive index variations in the $10^{-4}$ range. This envisages the development of high-resolution, greater PSHE-TD and low-cost dielectric material-based PSHE devices for commercial applications.

## Data Availability

The data may be obtained from the corresponding author (Y.M.) upon reasonable request.

## References

[1] Lotsch HKV (1968). Reflection and refraction of a beam of light at a plane interface. J. Opt. Soc. Am..

[2] Bliokh KY, Aiello A (2013). Goos-Hänchen and Imbert-Fedorov beam shifts: An overview. J. Opt..

[3] Bliokh KY, Bliokh YP (2004). Modified geometrical optics of a smoothly inhomogeneous isotropic medium: The anisotropy, Berry phase, and the optical Magnus effect. Phys. Rev. E.

[4] Onoda M, Murakami S, Nagaosa N (2004). Hall effect of light. Phys. Rev. Lett..

[5] Bliokh KY, Nori F (2012). Relativistic Hall effect. Phys. Rev. Lett..

[6] Bliokh KY, Bliokh YP (2007). Polarization, transverse shifts, and angular momentum conservation laws in partial reflection and refraction of an electromagnetic wave packet. Phys. Rev. E.

[7] Bliokh KY, Gorodetski Y, Kleiner V, Hasman E (2008). Coriolis effect in optics: Unified geometric phase and spin-hall effect. Phys. Rev. Lett..

[8] Hosten O, Kwiat P (2008). Observation of the spin hall effect of light via weak measurements. Science.

[9] Pang S, Wu S, Chen Z-B (2012). Weak measurement with orthogonal preselection and postselection. Phys. Rev. A.

[10] Luo H (2009). Spin hall effect of a light beam in left-handed materials. Phys. Rev. A.

[11] Jiang X (2018). Enhanced photonic spin hall effect with a bimetallic film surface plasmon resonance. Plasmonics.

[12] Xu W (2020). Giant photonic spin hall effect near the dirac points. Phys. Rev. A.

[13] Zhang W (2018). Photonic spin hall effect on the surface of anisotropic two-dimensional atomic crystals. Photon. Res.

[14] kim M (2019). Observation of enhanced optical spin hall effect in a vertical hyperbolic metamaterial. ACS Photon..

[15] Ling X-H, Luo H-L, Tang M, Wen SC (2012). Enhanced and tunable spin hall effect of light upon reflection of one-dimensional photonic crystal with a defect layer. Chin. Phys. Lett..

[16] Luo H, Zhou X, Shu W, Wen S, Fan D (2011). Enhanced and switchable spin hall effect of light near the brewster angle on reflection. Phys. Rev. A.

[17] Dong P, Cheng J, Da H, Yan X (2020). Controlling photonic spin hall effect in graphene-dielectric structure by optical pumping. New J. Phys..

[18] Wang H, He Y, Zhang J, Xu Y (2022). Highly sensitive refractive index sensor based on the lossy mode resonance enhanced photonic spin hall effect. JOSA B.

[19] Zhou X, Ling X (2016). Enhanced photonic spin hall effect due to surface plasmon resonance. IEEE Photon. J..

[20] Tan X-J, Zhu XS (2016). Enhancing photonic spin hall effect via long-range surface plasmon resonance. Opt. Lett..

[21] Gao C, Guo B (2017). Enhancement and tuning of spin Hall effect of light in plasma metamaterial waveguide. Phys. Plasmas.

[22] Hosseini A, Nejati H, Massoud Y (2007). Design of a maximally flat optical low pass filter using plasmonic nanostrip waveguides. Opt. Express.

[23] Goyal AK, Saini J (2020). Performance analysis of Bloch surface wave based sensor using transition metal dichalcogenides. Appl. Nanosci..

[24] Goyal AK, Pal S (2020). Design analysis of Bloch surface wave based sensor for haemoglobin concentration measurement. Appl. Nanosci..

[25] Goyal AK, Kumar A, Massoud Y (2022). Thermal stability analysis of surface wave assisted bio-photonic sensor. Photonics.

[26] Vinogradov AP (2010). Surface states in photonic crystals. Phys. Usp..

[27] Goyal AK, Saini J, Massoud Y (2023). Performance analysis of organic material assisted dynamically tunable excitation of optical tamm state. Opt. Quant. Electron..

[28] Lereu AL, Zerrad M, Passian A, Amra C (2017). Surface plasmons and Bloch surface waves: Towards optimized ultra-sensitive optical sensors. Appl. Phys. Lett..

[29] Goyal AK, Kumar A, Massoud Y (2022). Performance analysis of DAST material-assisted photonic-crystal-based electrical tunable optical filter. Curr. Comput.-Aided Drug Des..

[30] Tathfif I, Rashid KS, Yaseer AA, Sagor RH (2021). Alternative material titanium nitride based refractive index sensor embedded with defects: An emerging solution in sensing arena. Results Phys..

[31] Hassan MF, Sagor RH, Tathfif I, Rashid KS, Radoan M (2021). An optimized dielectric-metal-dielectric refractive index nanosensor. IEEE Sens..

[32] Rashid KS, Hassan MF, Yaseer AA, Tathfif I, Sagor RH (2021). Gas-sensing and label-free detection of biomaterials employing multiple rings structured plasmonic nanosensor. Sens. Bio-Sens. Res..

[33] Goyal AK, Kumar A, Massoud Y (2023). Performance analysis of heterostructure-based topological nanophotonic sensor. Sci. Rep..

[34] Rashid KS, Tathfif I, Yaseer AA, Hassan MF, Sagor RH (2021). Cog-shaped refractive index sensor embedded with gold nanorods for temperature sensing of multiple analytes. Opt. Express.

[35] Yeh P, Yariv A, Hong C-S (1977). Electromagnetic propagation in periodic stratified media I General theory. J. Opt. Soc. Am..

[36] Goyal, A. K., Pradhan, K. P., & Massoud, Y. Theoretical analysis of dielectric assisted Tamm mode excitation. In *2022 IEEE 22nd International Conference on Nanotechnology (NANO)* (2022).

[37] Luo H, Ling X, Zhou X, Shu W, Wen S, Fan D (2011). Enhancing or suppressing the spin Hall effect of light in layered nanostructures. Phys. Rev. A.

[38] Xiang Y, Jiang X, You Q, Guo J, Dai X (2017). Enhanced spin Hall effect of reflected light with guided-wave surface plasmon resonance. Photon. Res..

[39] Zhou X, Sheng L, Ling X (2018). Photonic spin hall effect enabled refractive index sensor using weak measurements. Sci. Rep..

[40] Goyal AK, Divyanshu D, Massoud Y (2023). Nanophotonic resonator assisted photonic spin Hall enhancement for sensing application. Sci. Rep..

[41] Liang C, Wang G, Deng D, Zhang T (2021). Controllable refractive index sensing and multi-functional detecting based on the spin hall effect of light. Opt. Express.

[42] Das D, Saini J, Goyal AK, Massoud Y (2023). Exponentially index modulated nanophotonic resonator for high-performance sensing applications. Sci. Rep..

[43] Goyal AK, Dutta HS, Pal S (2020). Development of uniform porous one-dimensional photonic crystal based sensor. Optik.

